# *In silico* study of colchicine resistance molecular mechanisms caused by tubulin structural polymorphism

**DOI:** 10.1371/journal.pone.0221532

**Published:** 2019-08-23

**Authors:** Harutyun Sahakyan, Narek Abelyan, Vahram Arakelov, Grigor Arakelov, Karen Nazaryan

**Affiliations:** 1 Department of Bioengineering, Bioinformatics and Molecular Biology, Russian-Armenian University, Yerevan, Armenia; 2 Laboratory of Computational Modeling of Biological Process, Institute of Molecular Biology, Yerevan, Armenia; Weizmann Institute of Science, ISRAEL

## Abstract

Starting from 1972, colchicine is known as the most useful drug for prevention of familial Mediterranean fever attacks. However, some patients do not respond to colchicine treatment, even taken in high doses. Despite the fact, that different hypotheses have been proposed, the molecular mechanisms of colchicine resistance are not completely clear. It is generally known, that colchicine binds β-tubulin and inhibits microtubules polymerization. The β-tubulin gene has SNPs, which lead to amino acid substitutions, and some of them are located in colchicine binding site (CBS). We have assumed, that this SNPs can affect tubulin-colchicine interaction and might be the reason for colchicine resistance. With this in mind, we modeled 7 amino acid substitutions in CBS, performed molecular dynamics simulations of tubulin-colchicine complex and calculated binding energies for every amino acid substitution. Thus, our study shows, that two amino acid substitutions in the β-tubulin, namely A248T and M257V, reduce binding energy for approximately 2-fold. Based on this, we assume, that these amino acid substitutions could be the reason for colchicine resistance. Thus, our study gives a new insight into colchicine resistance mechanism and provides information for designing colchicine alternatives, that could be effective for colchicine resistant patients.

## Introduction

Colchicine is a natural tricyclic alkaloid extracted from plants of Lily family *Colchicum autumnal* and *Gloriosa superba* in 1820. Colchicine inhibits microtubules polymerization, that is involved in a broad range of cellular processes, such as intracellular transport, cell division, chromosome segregation, regulation of cell polarity and maintenance of morphology [1].

Nowadays, the colchicine is actively used to suppress symptoms of familial Mediterranean fever (FMF) for prevention of amyloidosis and for treatment of some other diseases [2]. The therapeutic effect of colchicine in FMF is not completely clear. Colchicine is a cytostatic, which has antimitotic activity and inhibits polymerization of microtubules. Thus, there are suggestions, that colchicine reduces leukocyte division and chemotaxis, suppressing the inflammation process [3, 4].

According to different authors, up to 5% of FMF patients have colchicine resistance and do not respond to colchicine even in high dose treatment [5, 6]. Mechanisms of colchicine resistance in FMF remain unclear. There are no data about direct colchicine-pyrin interaction or direct interrelation between colchicine resistance and mutations in pyrin gene, which cause FMF. It is known, that the colchicine interacts with β-tubulin and inhibits tubulin polymerization or leads to microtubules depolymerization [7]. Genes of β-tubulin isotypes have several missense SNPs, that lead to amino acid substitutions in colchicine binding site. We assumed, that CBS structural polymorphisms, caused by these SNPs, may influence colchicine affinity for tubulin, decrease colchicine-tubulin binding energy and become the reason for colchicine resistance.

The human genome contains 10 tubulin genes, which encode different tubulin isotypes with differential tissue expression level [8]. Given the fact, that colchicine treatment of FMF primarily affects leukocytes activity, we considered SNPs of tubulin β1, encoded by TUBB1 gene, which is specific only for leukocytes [8, 9]. Moreover, earlier it was reported, that colchicine accumulates in neutrophils [10] and has the highest affinity for tubulin β1 (class VI) among all other tubulin isotypes [11]. Human TUBB1 gene has 7 missens SNPs (Table 1), which lead to amino acid substitution in CBS.

**Table 1:** SNPs in the tubulin β1 gene (TUBB1) are leading to amino acid substitution in CBS. Data are taken from dbSNP (https://www.ncbi.nlm.nih.gov/projects/SNP/snp_ref.cgi?geneId=81027).

Chromosome position	mRNA position	dbSNP rs# cluster id	dbSNP allele	Residue substitution	Codon position	AA position
59024143	985	rs1243106271	C→A	Ser [S]→Tyr [Y]	2	239
59024169	1011	rs148237574	G→A	Ala [A] → Thr[T]	1	248
59024184	1026	rs141721320	C→G	Leu [L]→ Val [V]	1	253
59024196	1038	rs759579888	A→G	Met [M]→Val [V]	1	257
59024197	1039	rs202095800	T→C	Met [M]→ Thr [T]	2	257
59024368	1210	rs763217749	C→T	Ala [A]→ Val [V]	2	314
59024373	1215	rs759314992	A→G	Ile [I]→Val [V]	1	316

In this study, we modeled tubulin structures with different amino acid substitutions in CBS and performed series of molecular dynamics simulations and binding energy calculations to confirm or deny the assumption, that structural polymorphism of β-tubulin may cause colchicine resistance.

## Results

**Fig 1:**
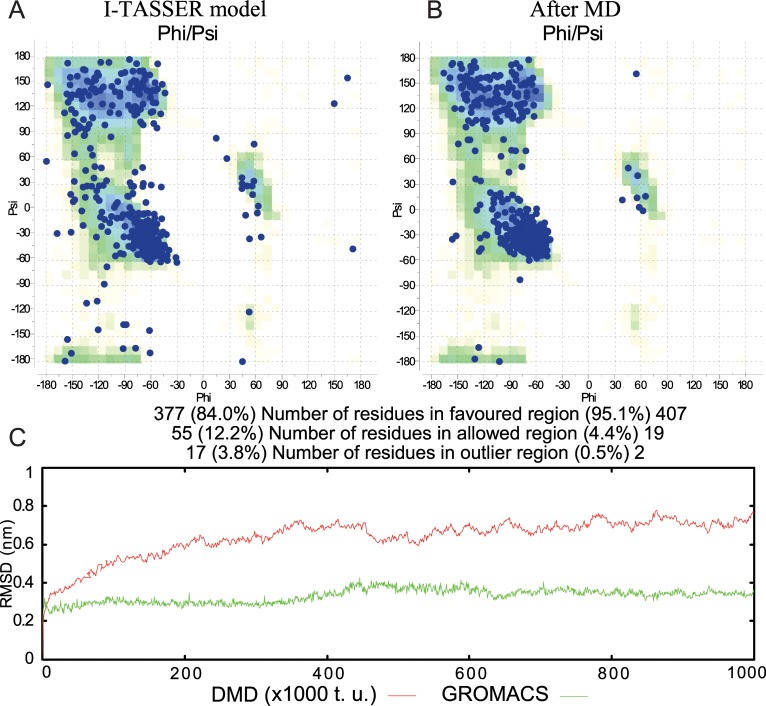
Comparison of modeled tubulin quality before and after MD. Ramachandran plots of tubulin β1 for I-TASSAR model (A) and after MD simulations (B). Root-mean-square deviation of MD and DMD.We performed MD simulation using piDMD (*discrete molecular dynamics*) and then continued with the same coordinates using GROMACS (C).

Since there is no human tubulin β1 crystallographic structure, we modeled it, using I-TASSER server [12]. For estimation of tubulin quality model, we built a Ramachandran plot, which represents dihedral angles of backbone energetically allowed regions. The predicted structure has 84.0% of amino acids in favored, 12.2% in allowed and 3.8% in outlier regions on Ramachandran plot (Fig 1A), while a good quality model is considered to be 90% or higher [13]. Further, we replaced β-subunit of the 4o2b crystallographic model with the obtained model and performed long-term molecular dynamics simulation, using piDMD program to ensure, that the modeled structure is stable, and the subunits do not diverge from each other. Hereafter, we added the colchicine and performed molecular dynamics simulation of tubulin-colchicine complex, using GROMACS with parameters, described in the Methods section. The coordinates of colchicine in CBS were taken from the above-mentioned crystallographic structure. We performed 1 μs molecular dynamics simulation to refine protein structure and to check the stability of the protein-ligand structure. Thereby, after MD simulations, the tubulin β1 modeled structure was refined on Ramachandran plot, where 95.1% of amino acids were located in favored, 4.4% in allowed and 0.5% in outlier regions (Fig 1B). During the MD protein-ligand complex was stable without any significant conformational changes in the protein structure. The colchicine in a binding site was stable as well.

Thus, RMSD fluctuation of tubulin dimer during DMD simulation was stabilized approximately after 4.5x10^5^ t. u. (time units) and did not exceed a range of 0.2 nm. Besides, tubulin-colchicine complex MD simulation was more stable and RMSD fluctuation did not exceed 0.1–0.15 nm throughout the simulation.

Thereby, once tubulin β1structure was modeled, we introduced amino acid substitutions, caused by the above-mentioned SNPs, to the tubulin structure, obtained after MD simulations. As a result, we got 7 new tubulin structures with different amino acids in CBS. All of the changed amino acids were located in CBS and could be involved in interaction with colchicine. Hereafter, we performed MD in every case to estimate possible changes in protein-ligand interaction mode and binding energy. Thus, MD simulations of tubulin-colchicine complexes were run for 300 ns with the same configurations, using GROMACS program suit. Then, we used g_mmpbsa program for the calculation of the binding free energy.

Herein, we identified 41 amino acids in tubulin β1 CBS within 6 Å cutoff range from colchicine. Energy decomposition per residue showed, that only some of them had a strong contribution to ligand binding. In general, colchicine interacts with tubulin β1 amino acids Ser-239, Leu-246, Ala-248, Leu-250, Lys-252, Leu-253, Asn-256, Met-257, Thr-312, Ala-314, Ile-316. According to g_mmpbsa calculations (See S1 Table for detailed information), colchicine binds to tubulin β1with -156 kJ/mol. As a matter of fact, tubulin-colchicine binding free energy may differ, depending on the methods of approaches to tubulin isotypes and crystallographic structures [11, 14]. The binding energy, calculated in our study, well fits the data provided by Majcher and his colleagues [15], which was calculated using Amber14 [16] and MM/PBSA [17] method.

**Fig 2:**
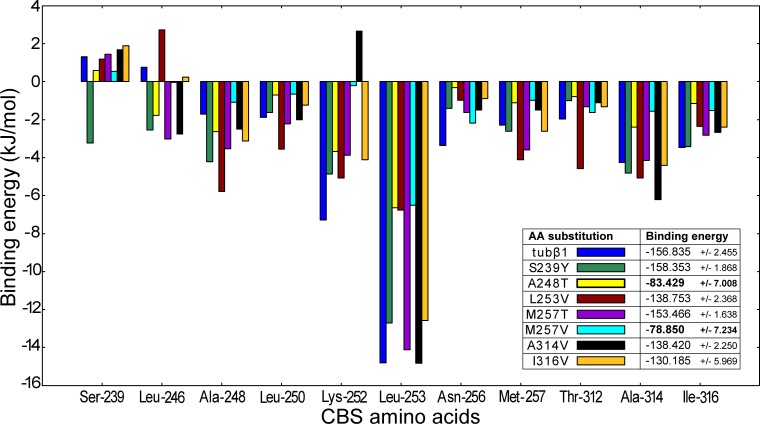
Decomposition of the estimated binding energies per residue for native tubulin β1and tubulin β1with different amino acid substitutions. The presented amino acids have the strongest contribution in colchicine binding. Interestingly, Leu-253 has the strongest impact, but the substitution of this amino acid (dark-red) just slightly reduces binding energy. Whereas, S248T (yellow) and M257V (cyan) also reduce Leu-253 contribution in binding energy. Moreover, these substitutions affect almost all CBS amino acids binding energy.

Interestingly, after amino acids substitution and MD simulations, colchicine undergoes minor conformational changes in the binding site (S1 Fig). Generally, these changes are associated with very flexible T7 and T5 loops in CBS [18]. Changes in binding energy are more obvious (Fig 2). Hence, the strongest contribution to colchicine binding has Leu-253 and substitution of this amino acid reduces binding energy, valine binds colchicine with -6.54 kJ/mol, while leucine in this position binds colchicine with -14,42 kJ/mol. However, other amino acids binding energy remains almost unchanged and total binding energy diminishes from -156.8 to -138.7 kJ/mol.

Nevertheless, two amino acid substitutions viz. A248T and M257V caused by rs148237574 and rs759579888 respectively, reduce binding energy by almost twofold. In these cases, in contrast to the L253V substitution, almost all other amino acids binding energies, which have a strong contribution to colchicine binding, are also reduced. Replacing alanine with threonine in 248 position decreases binding energy to -84 kJ/mol i.e. by ~ 54%. Apparently, in this case, tubulin-colchicine binding energy reduction occurs due to the replacement of hydrophobic amino acid with a polar one. Hydrophobic interactions play a crucial role in colchicine trimethoxybenzene ring (A ring) binding and have an important contribution in tubulin-colchicine binding energy [19]. However, in the case of M257V, any hydrophobic changes in CBS do not occur, since valine has a hydrophobic side chain as well as methionine. Nonetheless, stereochemical changes shift the colchicine outward from its binding pocket towards alpha subunit. As a result of the shift, the colchicine interacts with T5 loop of α-subunit, which is unusual. This change of tubulin-colchicine interaction mode most probably causes binding energy reduction.

Serine in the 239 position is specific for β-tubulin isoforms β1, β3 and β6 in contrast to other tubulin isotypes, that contain cysteine in this position [20]. It should be emphasized, that Ser-239 has a positive contribution in the binding energy, namely serine in 239 position does not attract the colchicine but pushes it away. Tyrosine in this position, in case of S239Y substitution, interacts with colchicine with a negative contribution in binding energy, and this is the only case, when we can observe the binding energy even superior to tubulin β1-colchicine binding energy.

## Discussion

Taking into consideration the fact, that there are many crystallographic structures of tubulin dimer, it is easy to obtain accurate tubulin isotypes and mutant structures, using homology modeling methods. Therefore, there are no doubts, related to the accuracy and stability of the modeled tubulin structure. The stability of these structures during MD simulations prove that.

According to our assumption, amino acid substitutions can be the cause colchicine resistance. Nevertheless, these SNPs must have a high frequency to be the sole reason for the colchicine resistance. However, we have insufficient information about the frequency of these SNPs. Do this SNPs have a high frequency in Mediterranean populations, where the FMF is more common? Unfortunately, there are not enough data about this, and we do not have an answer to this question for now. There are different hypotheses, explaining colchicine resistance mechanisms. Our study does not deny other colchicine resistance assumptions, such as polymorphism in ABCB1/ MDR1 gene encoding P-glycoprotein, level of CYP3A4, drug-drug interactions and others [21, 22, 23].

The study of Lidar et al., showed that colchicine concentration of leucocytes in colchicine resistance patients is twofold lower, than in colchicine tolerate group [6]. There is a supposition, that this can be caused by different polymorphisms in the ABCB1/MDR1 gene encoding P-glycoprotein. Nevertheless, Bezalel et al. failed to demonstrate this hypothesis [24]. We suppose, that the reason of high concentration of colchicine in lymphocytes can be the high affinity of colchicine to tubulin β1, which is specific for lymphocytes, and, respectively, in case of lower affinity the concentration of colchicine will be lower.

From our point of view, it is highly probable, that the colchicine resistance can be caused by different mechanisms, and the mechanism, described in this study, is only one of the reasons for colchicine resistance.

## Conclusion

In the current study, we considered the possibility of β-tubulin polymorphism in CBS to be a reason for colchicine resistance among FMF patients. Earlier, several studies have investigated the impact of beta-tubulin mutations on colchicine resistance, though the relation to FMF has not been studied [25,26]. For testing this hypothesis, we modeled tubulin β1that is specific for leucocytes, and introduced amino acid substitutions, that occur in CBS due to different SNPs. Further, to investigate the effect of these amino acid substitutions on tubulin-colchicine interaction we performed molecular dynamics simulations and calculated the binding free energy using MM-PBSA method. Thus, our study showed, that investigated substitutions led to minor changes in protein structure. However, in some cases, we observed binding energy reduction in comparison to the native tubulin β1-colchicine binding energy.

Based on our present in silico study, we propose that tubulin structural polymorphism may be one of the reasons for colchicine resistance. More specifically, A248T and M257V substitution may twofold weaken the binding affinity of colchicine to tubulin β1. Investigation of the role of structural polymorphism in colchicine resistance and colchicine affinity to tubulin is very important and MD simulations have significant contribution to this study. The information provided in this study constitutes bases for further *in vitro* investigations of colchicine resistance mechanisms and can be helpful for design an alternative therapeutic approach for colchicine-resistant FMF patients.

## Methods

### Structure modeling

Tubulin structure was modeled using I-TASSER [12]. We used human tubulin β1 sequence (UniProt ID: Q9H4B7) and tubulin-colchicine complex crystallographic structure (PDB ID: 4O2B) as a template for modeling. Further, we performed molecular dynamics simulations of tubulin β1 to refine the tubulin structure, obtained from I-TASSER. Thereafter, were introduced single amino acid substitutions, caused by SNPs, and minimized the complex structures using ICM program [27].

### Molecular dynamics simulation and binding free energy calculation

Molecular dynamics simulations were performed, using DMD [28] and GROMACS 2018.1 [29] program packages. We used piDMD for long-term MD simulation in order to check the stability of the obtained structure. For DMD simulations we used parameters, described in the original article [28]. Parameters, that were used for simulations with GROMACS, are described below. We used Amber99sb-ildn [30] force field for simulations, antechamber with general amber force field [31] for colchicine topology preparation and ACPYPE [32] for topology conversion to GROMACS compatible format. All simulations were performed without restraints, in explicit water environment, using TIP3P model [33]. We solvated tubulin-colchicine complex in a triclinic box with 111x80x71 sides Å, which contains ~80,000 atoms, and neutralized the system with the addition Na^+^ and Cl^-^ ions in 150 mM concentration. We minimized the system, using steepest descents energy minimization algorithm and performed system equilibration, using NVT and NPT ensembles simulations with 20 and 40 ns duration respectively. We performed MD simulations at temperature 310 K and at pressure 1 bar using V-rescale [34] algorithm for temperature coupling and Parrinello-Rahman [35] barostat for pressure coupling. The LINCS (LINear Constraint Solver) algorithm [36] for bond length constraining and the PME (Particle Mesh Ewald electrostatics) for long-range calculations [37] were used. The Coulomb and Lennard–Jones interactions were calculated using cutoff 1.0 nm. For all simulations we used 2 fs time step.

Binding free energies were calculated using g_mmpbsa program [38], with MM-PBSA method adopted for GROMACS. Thus, we used frames taken every 100 ps during the last 10 ns of MD simulation to estimate average binding energy. Therefore, we used 100 frames for MM-PBSA calculation with bootstrap analysis for each case.

## Supporting information

S1 Fig Analysis of the protein-ligand complexes stability. Root-mean-square deviation (A) and radius of gyration (B) of the tubulin β1 and its modeled analogs in complex with colchicine during MD. Root-mean-square fluctuation of CBS (C), the regions interacting with colchicine represented as protein secondary structure.
(EPS)

S1 Movie Visualized trajectory of the tubβI MD simulation. (MP4)

S2 Movie Visualized trajectory of the S239Y MD simulation. (MP4)

S3 Movie Visualized trajectory of the A248T MD simulation. (MP4)

S4 Movie Visualized trajectory of the L253V MD simulation. (MP4)

S5 Movie Visualized trajectory of the M257T MD simulation. (MP4)

S6 Movie Visualized trajectory of the M257V MD simulation. (MP4)

S7 Movie Visualized trajectory of the A314V MD simulation. (MP4)

S8 Movie Visualized trajectory of the I316V MD simulation. (MP4)

S1 Table Binding energies estimated with *g_mmpbsa* for tubulin β1 and its modeled analogs with colchicine. Energies are given in kJ/mol with standard error.
(DOCX)

S2 Table Hydrogen bonds analyze. H-bonds donors (D), acceptors (A) and occupancy (O) during simulation.
(DOCX)

## Abbreviations

CBS: colchicine binding site
FMF: familial Mediterranean fever
tubulin β1: tubulin beta-1
MD: molecular dynamics
DMD: discrete molecular dynamics
SNP: single nucleotide polymorphism
