# Supplementary material for: In silico study of colchicine resistance molecular mechanisms caused by tubulin structural polymorphism
Source: PLoS One. 2019 Aug 23;14(8):e0221532. doi: 10.1371/journal.pone.0221532 (PMC6707608; doi:10.1371/journal.pone.0221532)
Supplement: S1 Table — Energies are given in kJ/mol with standard error. (DOCX) [file pone.0221532.s010.docx]

**Table S1**. **Binding energies** estimated with *g_mmpbsa* for tubulin β1 and its modeled analogs with colchicine. Energies are given in kJ/mol with standard error.

|  | tubβI | S239Y | A248T | L253V | M257T | M257V | A314V | I316V |
| --- | --- | --- | --- | --- | --- | --- | --- | --- |
| van der Waal energy | -227.372 +/- 1.687 | -252.263 +/- 3.130 | -132.554 +/- 10.634 | -238.779 +/- 1.903 | -235.168 +/- 1.722 | -130.924 +/- 11.133 | -237.268 +/- 1.602 | -192.356 +/- 7.712 |
| Electrostatic energy | -32.372 +/- 1.786 | -50.449 +/- 2.904 | -38.663 +/- 3.716 | -17.732 +/- 2.710 | -49.912 +/- 2.384 | -14.971 +/- 1.858 | -44.407 +/- 1.861 | -30.033 +/- 2.542 |
| Polar solvation energy | 124.113 +/- 2.759 | 165.446 +/- 2.884 | 100.943 +/- 8.667 | 139.618 +/- 2.285 | 152.445 +/- 2.163 | 80.482 +/- 6.552 | 164.772 +/- 2.613 | 111.610 +/- 5.392 |
| SASA energy | -21.059 +/- 0.099 | -21.113 +/- 0.150 | -12.857 +/- 0.892 | -21.937 +/- 0.140 | -20.832 +/- 0.109 | -12.867 +/- 0.922 | -21.500 +/- 0.114 | -19.555 +/- 0.723 |
| **Binding energy** | **-156.835 +/- 2.455** | **-158.353 +/- 1.868** | **-83.429 +/- 7.008** | **-138.753 +/- 2.368** | **-153.466 +/- 1.638** | **-78.850 +/- 7.234** | **-138.420 +/- 2.250** | **-130.185 +/- 5.969** |
