# Supplementary material for: In silico study of colchicine resistance molecular mechanisms caused by tubulin structural polymorphism
Source: PLoS One. 2019 Aug 23;14(8):e0221532. doi: 10.1371/journal.pone.0221532 (PMC6707608; doi:10.1371/journal.pone.0221532)
Supplement: S2 Table — H-bonds donors (D), acceptors (A) and occupancy (O) during simulation. (DOCX) [file pone.0221532.s011.docx]

**Table S2. Hydrogen bonds analyze.** H-bonds donors (D), acceptors (A) and occupancy (O) during simulation.

| tub β1 | | | S239Y | | | A248T | | | L253V | | |
| --- | --- | --- | --- | --- | --- | --- | --- | --- | --- | --- | --- |
| D | A | O | D | A | O | D | A | O | D | A | O |
| THR179 | COL | 0.70% | THR179 | COL | 3.78% |  | - | - | THR179 | COL | 0.66% |
| - | - | - | VAL181 | COL | 0.20% | VAL181 | COL | 0.13% | - | - | - |
| SER239 | COL | 3.48% | - | - | - | SER239 | COL | 0.13% | SER239 | COL | 2.26% |
| ASP249 | COL | 0.46% | ASP249 | COL | 1.20% | ASP249 | COL | 0.53% | ASP249 | COL | 0.13% |
| ASN347 | COL | 0.93% | ASN347 | COL | 4.58% | ASN347 | COL | 2.39% | ASN347 | COL | 0.13% |
| LYS350 | COL | 5.34% | LYS350 | COL | 5.38% | LYS350 | COL | 4.26% | LYS350 | COL | 4.26% |
| COL | ASN247 | 0.23% | COL | ASN247 | 0.20% | - | - | - | COL | ASN247 | 0.80% |
| M257T | | | M257V | | | A314V | | | I316V | | |
| D | A | O | D | A | O | D | A | O | D | A | O |
| THR179 | COL | 1.06% | - | - | - | THR179 | COL | 4.65% | THR179 | COL | 2.42% |
| - | - | - | - | - | - | VAL181 | COL | 0.13% | VAL181 | COL | 0.13% |
| SER239 | COL | 1.60% | - | - | - | - | - | - | SER239 | COL | 7.18% |
| ASP249 | COL | 1.20% | ASP249 | COL | 0.40% | ASP249 | COL | 2.39% | ASP249 | COL | 0.27% |
| ASN347 | COL | 0.80% | - | - | - | ASN347 | COL | 1.60% | ASN347 | COL | 0.66% |
| LYS350 | COL | 2.93% | LYS350 | COL | 1.20% | LYS350 | COL | 6.91% | LYS350 | COL | 3.99% |
| COL | ASN247 | 0.13% | THR179 | ASN247 | 0.13% | THR179 | ASN247 | 0.13% | COL | ASN247 | 0.27% |
